# Safety and effectiveness of remdesivir in hospitalized patients with COVID-19 and severe renal impairment: experience at a large medical center

**DOI:** 10.1080/07853890.2024.2361843

**Published:** 2024-06-03

**Authors:** Hsuan-Yu Chang, Chia-Chen Hsu, Li-Fang Hu, Chian-Ying Chou, Yuh-Lih Chang, Chih-Chia Lu, Li-Jen Chang

**Affiliations:** a Department of Pharmacy, Taipei Veterans General Hospital, Taipei, Taiwan; b Department of Pharmacy, College of Pharmaceutical Sciences, National Yang Ming Chiao Tung University, Taipei, Taiwan; c Institute of Pharmacology, College of Medicine, National Yang Ming Chiao Tung University, Taipei, Taiwan

**Keywords:** COVID-19, remdesivir, severe renal impairment, acute kidney injury

## Abstract

**Background:**

Literature on the safety of remdesivir in hospitalized COVID-19 patients with severe renal impairment is limited. We aimed to investigate the safety and effectiveness of remdesivir in this population.

**Methods:**

We conducted a retrospective cohort study of adult hospitalized COVID-19 patients who received remdesivir between April 2022 and October 2022. Outcomes were compared between estimated glomerular filtration rate (eGFR) <30 mL/min/1.73 m^2^ and ≥30 mL/min/1.73 m^2^ groups. The primary safety outcomes were acute kidney injury (AKI) and bradycardia, while the primary effectiveness outcomes included mortality in COVID-19-dedicated wards and hospital mortality. Secondary outcomes included laboratory changes, disease progression, and recovery time.

**Results:**

A total of 1,343 patients were recruited, with 307 (22.9%) in the eGFR <30 group and 1,036 (77.1%) in the eGFR ≥30 group. Patients with an eGFR <30 had higher risks of AKI (adjusted hazard ratio [aHR] 2.92, 95% CI 1.93–4.44) and hospital mortality (aHR 1.47, 95% CI 1.06–2.05) but had comparable risks of bradycardia (aHR 1.15, 95% CI 0.85–1.56) and mortality in dedicated wards (aHR 1.43, 95% CI 0.90–2.28) than patients with an eGFR ≥30. Risk of disease progression was higher in the eGFR <30 group (adjusted odds ratio 1.62, 95% CI 1.16–2.26). No difference between the two groups in laboratory changes and recovery time.

**Conclusions:**

Hospitalized COVID-19 patients receiving remdesivir with severe renal impairment had an increased risk of AKI, hospital mortality, and COVID-19 disease progression compared to patients without severe renal impairment.

## Introduction

The Coronavirus Disease (COVID-19) has led to 770 million infections and 7 million deaths worldwide [1]. Chronic kidney disease (CKD) imposes a substantial global burden, with an estimated prevalence of 9.1%, reflecting a 29.3% increase over the past three decades [2]. Individuals with CKD have an increased susceptibility to severe COVID-19 and an increased risk of mortality [3–5].

Remdesivir – an adenosine triphosphate analog and RNA-dependent RNA polymerase inhibitor – stands as the first antiviral drug to receive emergency use authorization from both the United States and European Union [6,7]. Notably, patients with an estimated glomerular filtration rate (eGFR) below 30 mL/min/1.73 m^2^ were excluded from the registrational trials of remdesivir [8], rendering its usage not suitable for this group [9]. Animal studies on repeat-dose toxicity have revealed the transient presence of the metabolite GS-704277, potentially contributing to adverse renal events following remdesivir administration [10]. Additionally, caution in patients with renal impairment rose from concerns about the accumulation of the excipient sulfobutyl ether beta-cyclodextrin (SBECD) [2, 11,12]. However, despite these considerations, remdesivir is frequently employed in clinical settings involving patients with compromised renal function.

The majority of currently published studies examining remdesivir use in patients with an eGFR <30 encompass observational investigations, case reports, and case series studies [13]. Virtually all studies indicate that remdesivir is relatively safe and well-tolerated among patients with severe renal impairment [14–21]. It must be noted, however, that these studies are subject to various research limitations, including small sample sizes, absence of comparison groups, and failure to adequately control for potential confounding variables, which collectively contribute to the limited reliability of their findings. In contrast, studies utilizing the US FDA Adverse Events Reporting System (FAERS) and international pharmacovigilance post-marketing databases (VigiBase) have reported a higher incidence of kidney injury associated with remdesivir [22–24].

There is an ongoing debate concerning the safety of remdesivir in patients with severe renal impairment. Hence, this study aimed to investigate the safety and effectiveness among hospitalized COVID-19 patients receiving remdesivir, stratified by their eGFR: those with an eGFR <30 mL/min/1.73 m^2^ and those with an eGFR ≥30 mL/min/1.73 m^2^.

## Methods

### Ethics statement

This study received approval from the Institutional Review Board at Taipei Veterans General Hospital (TPEVGH IRB; No. 2023-04-009AC) and was carried out in accordance with the principles outlined in the Declaration of Helsinki. As the investigation presented little risk to the participants and did not involve any procedures, the TPEVGH IRB waived the need for written informed consent from the patients.

### Study design and data source

This single-center, retrospective cohort study was conducted at Taipei Veterans General Hospital, one of the largest medical centers in Taiwan. The hospital allows more than 2.5 million outpatient visits for 1.1 million patients each year. Data were collected by reviewing electronic medical records from the study hospital’s information system.

### Study population

We identified all eligible patients aged 20 years and older who were confirmed COVID-19-positive by real-time polymerase chain reaction and received remdesivir for the first time for the treatment of COVID-19 at the COVID-19-dedicated wards between April 01, 2022, and October 31, 2022. Noteworthily, the research period coincided with the epidemic period of the Omicron variant strains. Remdesivir as a lyophilized powder (Veklury®) was provided free of charge by the Taiwan Centers for Disease Control (CDC) after review and approval by epidemic prevention physicians. Patients with any of the risk factors for severe illness (e.g. age ≥65 years, diabetes, CKD, cardiovascular disease, chronic pulmonary disease), not on oxygen, and within 7 days of onset of illness, are treated for 3 days. For patients with oxygen saturation less than 94% without oxygen therapy or, in severe cases, requiring oxygen therapy, the treatment period is 5 days. Patients were administered 200 mg of remdesivir on the first day, followed by 100 mg/day for 3 to 5 days.

Patients were excluded if one of the following events existed: had a regimen change from a 3-day to a 5-day regimen; still hospitalized on December 31, 2022; alanine aminotransferase (ALT) levels greater than 5 times the upper limit of the normal (ULN) range prior to the index date; had human immunodeficiency virus infection or were pregnant; whose indications did not comply with the regulations of Taiwan Centers for Disease Control; who had incomplete important information (e.g. age, sex). The index date is defined as the date of receiving the first dose of remdesivir. Patients whose remdesivir regimen was changed from 3 to 5 days were excluded from this study because it was not available to know whether the additional days were subsequently due to poor remdesivir efficacy or disease worsening.

### Baseline covariates

The baseline characteristics collected included age, sex, body mass index, comorbidities, Charlson comorbidity index (CCI) [25], smoking status, COVID-19 disease severity, cycle threshold value of SARS-CoV-2, concomitant nephrotoxic drugs, adjuvant COVID-19 therapies, antibiotics, number of days before starting remdesivir after diagnosing of COVID-19, duration of remdesivir treatment (3-day or 5-day regimen), oxygen requirement, and laboratory data (ALT, total bilirubin, eGFR, serum creatinine), heart rate, and COVID-19 vaccine doses. COVID-19 disease severity was classified as mild to moderate, severe, and critical according to the guidelines of the Taiwan Center for Disease Control [26]. eGFR was calculated using the Modification of Diet in Renal Disease (MDRD) study equation [27].

### Ascertainment of exposure and outcomes

All qualified participants were categorized into two groups: eGFR less than 30 mL/min/1.73 m^2^ and eGFR greater than or equal to 30 mL/min/1.73 m^2^ group. Patients on dialysis were assigned to the eGFR < 30 group.

Primary safety outcomes were acute kidney injury (AKI) and bradycardia. AKI is defined as an increase in serum creatinine (SCr) of 0.3 mg/dL during the administration of remdesivir 48 h after the end of treatment or an increase in SCr exceeding 1.5 times the baseline value during the administration of remdesivir 7 days after the end of treatment according to Kidney Disease: Improving Global Outcomes (KDIGO) staging [28]. Patients who were already undergoing dialysis were excluded from this analysis due to the potential instability of their SCr levels, which could result in false AKI outcomes. Bradycardia is defined as a heart rate of fewer than 60 beats per minute (bpm), except for patients whose heart rate was originally under 60 bpm [29]. Heart rate was measured by an electronic blood pressure monitor. Secondary safety outcomes were changes in hepatic and renal laboratory data, including ALT, total bilirubin, eGFR, and SCr. Laboratory data were monitored from the index date to 7 days after the end of remdesivir treatment, and the worst value was recorded. Early discontinuation of remdesivir due to its side effects was also recorded.

Primary effectiveness outcomes were mortality in COVID-19-dedicated wards and mortality in the hospital. Mortality in dedicated wards was assessed from the index date to the occurrence of the event or the discharging from the dedicated wards, and mortality in the hospital from the index date to the occurrence of the event or discharge. The rules for transferring in and out of the dedicated wards were in accordance with Taiwan CDC regulations [30,31]. Patients diagnosed with COVID-19 may be admitted to a dedicated ward, except those who are asymptomatic or mildly ill, under 65 years of age, and able to function independently. If the patient’s fever has been suppressed for at least one day, symptoms have resolved, respiratory tests are PCR negative or the cycle threshold value of SARS-CoV-2 ≥ 30, and the attending physician evaluates the patient as suitable for transfer, the patient may be transferred out of the dedicated ward.

Secondary effectiveness outcomes were the progression of COVID-19 disease and the recovery time from COVID-19. The progression of COVID-19 disease is defined as the requirement for oxygen in patients receiving the 3-day remdesivir regimen or the need for advanced equipment to deliver supplemental oxygen in patients receiving the 5-day remdesivir regimen. Monitoring was conducted from the date of remdesivir discontinuation until the patients were transferred out of the dedicated wards. The order of equipment advancement is a nasal cannula, simple mask, venturi mask, nonrebreather mask, high-flow nasal cannula, bi-level positive airway pressure, and mechanical ventilator. Patients who received a 5-day regimen and were on an invasive ventilator at the index date were not included in the analysis of this outcome because the outcome did not occur in them. The recovery time from COVID-19 included the length of stay in dedicated wards and the length of oxygen requirement. The former measured the number of days from the index date to the date of discharge from the dedicated wards, while the latter measured the number of days from the index date to the removal of supplemental oxygen for patients following a 5-day regimen.

### Statistical analyses

For baseline characteristics, categorical variables were presented as frequencies and percentages and compared using the chi-square test or Fisher’s exact test with P values. Continuous variables were summarized as mean with standard deviations (SD) for normally distributed data or median with interquartile range (IQR) for non-normally distributed data. The statistical significance of differences between the two groups was determined using the student’s t-test or Mann-Whitney U test with *p* values, respectively.

For primary outcomes, the incidence rate was calculated as events per 1,000 patient-days. The Kaplan-Meier curve was used to estimate the event-free survival, and the log-rank test was used to compare the curves between groups. Cox proportional hazard models were used to compare primary outcomes between groups, in which hazard ratios (HR) with 95% CIs were reported. For secondary outcomes, multivariate logistic regressions or multivariate linear regressions were used, in which odds ratios (OR) or effect sizes (coefficient β) with 95% CIs were reported. All outcomes were adjusted for relevant confounders, as shown in Table 1. Statistical significance was determined at a 2-sided α level of 0.05.

**Table 1.** Baseline characteristics of study population.

Variables	eGFR < 30 mL/min/1.73 m^2^ *N* = 307	eGFR ≥ 30 mL/min/1.73 m^2^ *N* = 1,036	*p* value
Age, yr, median (IQR)	79.3 (69.0–89.9)	81.2 (67.3–90.7)	0.727
Male, n (%)	181 (59.0)	680 (65.6)	**0.038**
BMI, kg/m^2^, median (IQR)	22.41 (20.4–25.4)	22.29 (19.8–25.2)	0.407
Comorbidity, n (%)			
CCI, median (IQR)	4.0 (3.0–6.0)	3.0 (2.0–4.0)	**<0.001**
Hypertension	225 (73.3)	585 (56.6)	**<0.001**
Diabetes mellitus	143 (46.6)	300 (29.0)	**<0.001**
Cardiovascular disease	159 (51.8)	394 (38.0)	**<0.001**
Chronic lung disease	31 (10.1)	105 (10.1)	1.000
Chronic liver disease	33 (10.7)	174 (16.8)	**0.013**
Cancer	95 (30.9)	326 (31.5)	0.918
Cerebrovascular accident	56 (18.2)	225 (21.5)	0.217
Hemodialysis	122 (39.7)	0 (0.0)	
Smoking, n (%)			0.473
Non-smoker	253 (82.4)	872 (84.2)	
Ever smoker	31 (10.1)	106 (10.2)	
Current smoker	23 (7.5)	58 (5.6)	
COVID-19 disease severity (n, %)			**0.015**
Mild to moderate	93 (30.3)	314 (30.3)	
Severe	185 (60.3)	670 (64.7)	
Critical	29 (9.4)	52 (5.0)	
Ct value, cycle, median (IQR)	15.7 (13.4–19.3)	15.2 (13.0–18.7)	0.181
Concomitant nephrotoxic drug, n (%)	0.0 (0.0–1.0)	0.0 (0.0–1.0)	**0.022**
Vancomycin	14 (4.6)	4 (0.4)	**<0.001**
Aminoglycoside	1 (0.3)	1 (0.1)	0.405
Acyclovir	1 (0.3)	14 (1.4)	0.214
TMP/SMX	9 (2.9)	37 (3.6)	0.717
Amphotericin B	2 (0.7)	0 (0.0)	0.052
ACEIs/ARBs	70 (22.8)	217 (20.9)	0.537
Loop/thiazide diuretics	67 (21.8)	168 (16.2)	**0.029**
Tacrolimus/cyclosporine	15 (4.9)	20 (1.9)	**0.008**
NSAIDs	2 (0.7)	58 (5.6)	**<0.001**
Voriconazole	0 (0.0)	3 (0.3)	1.000
Adjuvant COVID-19 therapies, n (%)			
Dexamethasone	172 (56.0)	553 (53.4)	0.452
Tocilizumab	19 (6.2)	33 (3.2)	**0.026**
Antibiotics, n (%)	209 (68.1)	602 (58.1)	**0.002**
No. of days to start RDV after diagnosing, median (IQR)	2.0 (1.0-3.0)	2.0 (1.0-3.0)	0.602
5-Day RDV regimen, n (%)	214 (69.7)	722 (69.7)	1.000
Oxygen requirement, n (%)			**0.008**
Room air	118 (38.4)	399 (38.5)	
Nasal cannula	115 (37.5)	478 (46.1)	
Simple mask	0 (0.0)	2 (0.2)	
Venturi mask	16 (5.2)	47 (4.5)	
Nonrebreather mask	19 (6.2)	40 (3.9)	
High-flow nasal cannula	5 (1.6)	12 (1.2)	
Bi-level positive airway pressure	5 (1.6)	6 (0.6)	
Mechanical ventilator	29 (9.4)	52 (5.0)	
Laboratory data, median (IQR)			
ALT (U/L)	16.0 (10.0–28.0)	18.0 (12.0–29.0)	**0.001**
Total bilirubin (mg/dL)	0.4 (0.3–0.6)	0.5 (0.3–0.7)	**0.002**
eGFR (mL/min/1.73 m^2^)	15.0 (8.0–24.0)	70.0 (51.0–95.0)	**<0.001**
SCr (mg/dL)	3.6 (2.4–5.7)	1.0 (0.7–1.2)	**<0.001**
Heart rate, bpm, median (IQR)	73.0 (66.0–84.0)	75.0 (66.0–85.0)	0.158
COVID-19 vaccine doses, median (IQR)	2.0 (0.0–3.0)	2.0 (0.0–3.0)	0.131

Bold values indicate statistical significance at the *p* < 0.05 level. ACEIs/ARBs: angiotensin converting enzyme inhibitors / angiotensin receptor blockers, ALT: alanine transaminase, bpm: beats per minute, BMI: body mass index, COVID-19: coronavirus disease 2019, CCI: Charlson Comorbidity Index, Ct: cycle threshold, eGFR: estimated glomerular filtration rate, calculated using MDRD formula, IQR: interquartile range, NSAIDs: non-steroidal anti-inflammatory drug, RDV: remdesivir, SCr: serum creatinine, TMP/SMX: trimethoprim/sulfamethoxazole.

All data extracted from electronic medical records were logged into a formatted Microsoft Excel (Microsoft Corporation) template. These data were categorized and tabulated for further analysis. Statistical analyses were performed using the SPSS software (version 24; IBM Corp., Armonk, NY, USA) and the STATA software (version 17; StataCorp., College Station, Brazos, USA).

### Sensitivity analyses

We performed sensitivity analyses to test the robustness of our main results. The Chronic Kidney Disease Epidemiology Collaboration (CKD-EPI) formula and the Cockcroft-Gault (CG) formula are widely utilized in clinical practice for calculating eGFR [32–34]. Therefore, we first employed these two formulas to calculate eGFR to validate the obtained results. Second, considering the variations in AKI definitions across different guidelines, we employed the Acute Dialysis Quality Initiative (ADQI) staging and the Acute Kidney Injury Network (AKIN) staging to define the outcome of AKI [35,36].

## Results

**Figure 1.**
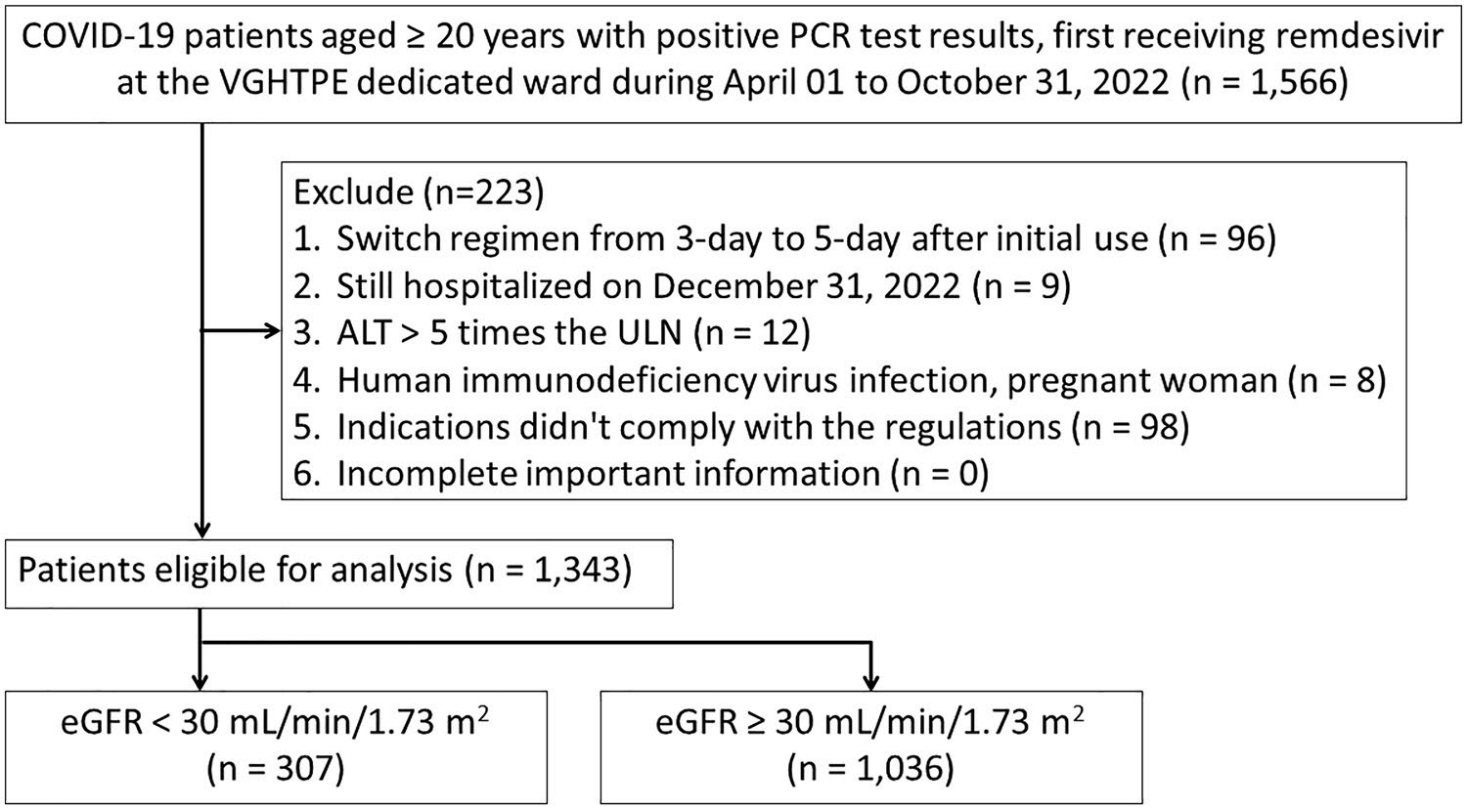
Flow diagram summarizing the process of enrollment.

A total of 1,343 patients were enrolled, of whom 307 (22.9%) were in the eGFR < 30 mL/min/1.73 m^2^ group and 1,036 (77.1%) in the eGFR ≥ 30 mL/min/1.73 m^2^ group. The flow chart for the enrollment of the study population is depicted in Figure 1.

### Baseline patient characteristics

The baseline characteristics of the study population are presented in Table 1. For the eGFR < 30 and eGFR ≥ 30 groups, the median (IQR) age was 79.3 (69.0–89.9) and 81.2 (67.3–90.7) years, respectively. There were more males in the eGFR ≥ 30 group (eGFR < 30: 59.0% vs eGFR ≥ 30: 65.6%, *p* = 0.038). Compared with the eGFR ≥ 30 group, the eGFR < 30 group had higher CCI scores and a prevalence of hypertension, diabetes mellitus, and chronic heart disease but a lower prevalence of chronic liver disease. The two groups had differences in COVID-19 disease severity, concomitant nephrotoxic drug, tocilizumab use, antibiotics use, oxygen requirement, and baseline laboratory data.

### Safety outcomes

Regarding the safety outcome, 39 (39/185 = 21.1%) of the eGFR < 30 group and 76 (76/1,036 = 7.3%) of the eGFR ≥ 30 group experienced acute kidney injury (incidence rate 28.6 versus 9.1 events per 1000 patient-days). The eGFR <30 group were more associated with a higher risk of acute kidney injury (aHR 2.92, 95% CI 1.93–4.44) than the eGFR ≥ 30 group. There was no difference in the bradycardia events and the changes in liver and kidney laboratory data between the two groups. Early discontinuation of remdesivir was observed in 7 (2.3%) and 9 patients (0.9%) in the eGFR < 30 group and the eGFR ≥ 30 group, respectively. The causes were bradycardia (5 in the eGFR < 30 group and 7 in the eGFR ≥ 30 group) and elevated liver enzymes (2 in the eGFR < 30 group and 2 in the eGFR ≥ 30 group).

### Effectiveness outcomes

**Figure 2.**
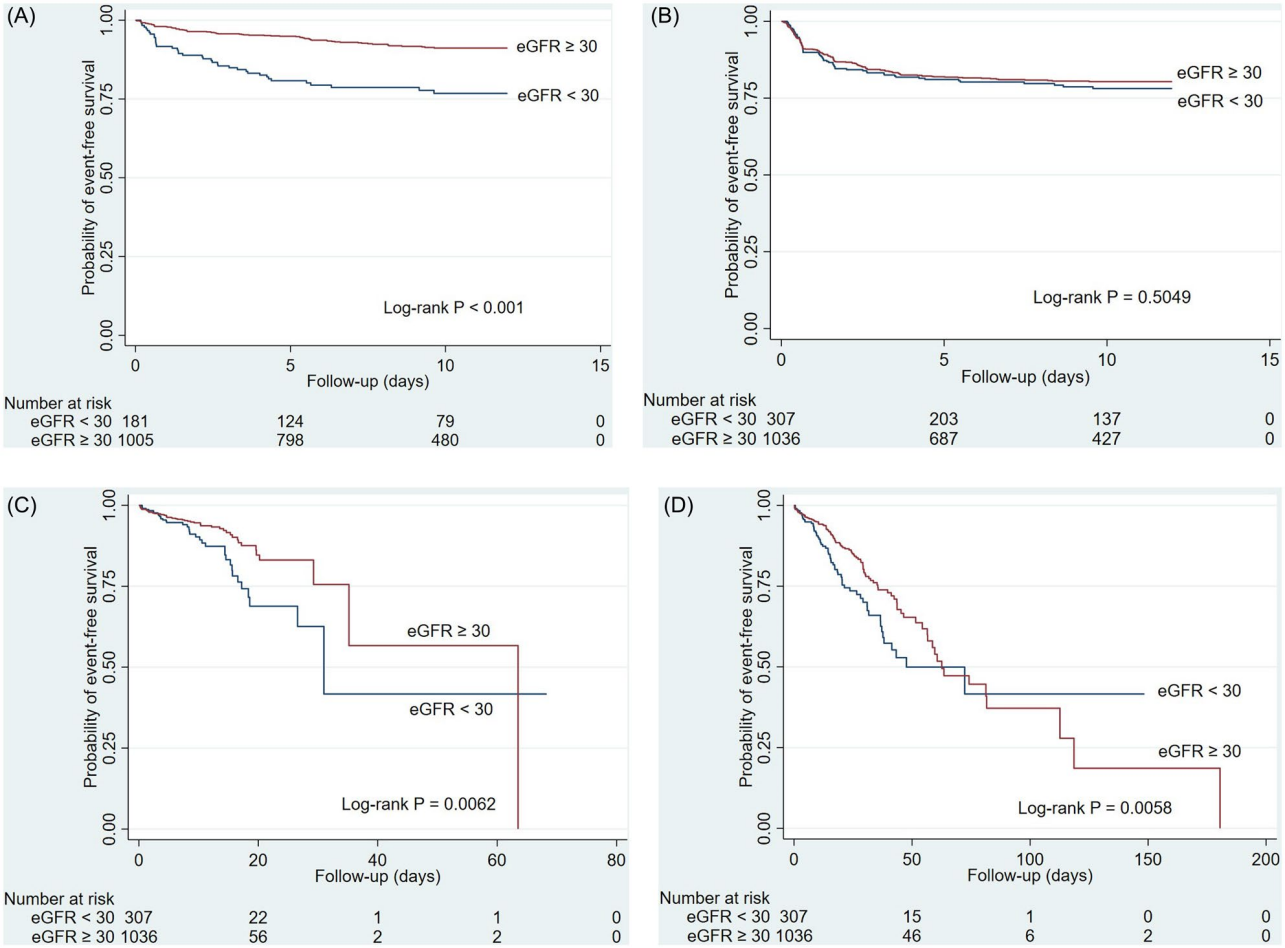
Kaplan-Meier survival curves of the COVID-19 cohort receiving remdesivir (A) acute kidney injury (B) bradycardia (C) mortality in COVID-19 dedicated ward (D) Mortality in hospital.

The incidence rates and adjusted hazard ratios (aHR) of outcomes are expressed in Table 2, the ORs/β are shown in Table 3, and the Kaplan-Meier survival curves of outcomes are depicted in Figure 2. The incidence rate of mortality in dedicated wards was 12.6 and 6.9 per 1000 patient-days for eGFR < 30 and eGFR ≥ 30 groups, with a considerably higher risk of mortality in dedicated wards between the groups (log-rank *p =* 0.0062). However, eGFR < 30 was not associated with a higher risk of mortality in dedicated wards (aHR 1.43, 95% CI 0.90–2.28) after adjustment for potential confounders in multivariate Cox regression analysis. The in-hospital mortality rate for all patients was 14.1% (190/1343). The incidence rate of mortality in the hospital was 11.6 and 7.5 per 1000 patient-days for eGFR < 30 and eGFR ≥ 30 groups, with a considerably higher risk of mortality in dedicated wards between the groups (log-rank *p =* 0.0058). After adjustment, eGFR < 30 was associated with a higher risk of mortality in the hospital [aHR 1.47, 95% CI 1.06–2.05).

**Table 2.** Incidence rates and hazard ratios for primary outcomes.

Outcome	eGFR < 30 *N* = 307			eGFR ≥ 30 *N* = 1,036			Adjusted HR (95% CI)
	Events	PD	IR	Events	PD	IR	
Acute kidney injury	39^a^	1365	28.6	76^b^	8348	9.1	**2.92 (1.93–4.44)** ^c^
Bradycardia	63	2245	28.1	192	7465	25.7	1.15 (0.85–1.56)^d^
Mortality in dedicated wards	36	2858	12.6	63	9140	6.9	1.43 (0.90–2.28)^e^
Mortality in hospital	64	5498	11.6	126	16785	7.5	**1.47 (1.06–2.05)** ^e^

Bold values indicate statistical significance at the *p* < 0.05 level. CI: confidence interval, eGFR: estimated glomerular filtration rate in mL/min/1.73 m2, calculated using MDRD formula, HR: hazard ratio, IR: Incidence rate per 1000-patient days, PD: Patient-days.

^a^ Excluded 122 patients on dialysis and 4 patients with data missing. Total number of observers was 181 in eGFR < 30 group.

^b^ Excluded 31 patients with data missing. Total number of observers was 1005 in eGFR ≥ 30 group.

^c^ Adjusted for age, sex, COVID-19 disease status, vaccine, CCI score, the 10 nephrotoxic drugs listed in Table 1.

^d^ Adjusted for age, sex, COVID-19 disease status, vaccine, CCI score, hypertension, diabetes mellitus, chronic heart disease, cerebrovascular accident.

^e^ Adjusted for age, sex, COVID-19 disease status, vaccine, CCI score, hypertension, diabetes mellitus, chronic heart disease, chronic lung disease, chronic liver disease, cancer, cerebrovascular accident, smoke, oxygen requirement.

**Table 3.** Secondary outcomes.

	eGFR < 30 *N* = 307	eGFR ≥ 30 *N* = 1,036	aOR/β (95% CI)	*p* value
Laboratory data change, median (IQR)				
ALT^a^	3.00 (-1.00–12.00)	4.00 (-1.00–15.25)	7.20 (-36.13–50.51)^b^	0.745
Total bilirubin^c^	0.03 (-0.11–0.17)	0.02 (-0.14–0.20)	−0.08 (-0.34–0.18)^b^	0.549
eGFR^d^	0.00 (-3.00–4.00)	1.00 (-10.50–11.00)	1.99 (-1.58–2.26)^e^	0.273
SCr^f^	−0.06 (-0.56–0.40)	−0.01 (-0.13–0.11)	0.08 (-0.04–0.19)^e^	0.180
Progression of COVID-19 disease, n/N (%)^g^	76/278 (27.3)	175/984 (17.8)	**1.62 (1.16–2.26)** ^h^	**0.004**
3-day remdesivir regimen	18/93 (19.4)	37/314 (11.8)	1.34 (0.68–2.61)^h^	0.457
5-day remdesivir regimen^g^	58/185 (31.4)	138/670 (20.6)	**1.73 (1.17–2.55)** ^h^	**0.006**
Length of stay in dedicated ward, median (IQR)	9.0 (7.0–15.0)	9.0 (7.0–14.0)	−0.08 (-0.97–0.81)^i^	0.863
Length of oxygen requirement, median (IQR)^j^	8.6 (3.4–17.1)	6.2 (2.6–13.7)	0.34 (-1.92–2.60)^h^	0.769

Bold values indicate statistical significance at the *p* < 0.05 level. ALT: alanine transaminase, aOR: adjusted odds ratio, CI: confidence interval, eGFR: estimated glomerular filtration rate in mL/min/1.73 m^2^, calculated using MDRD formula, IQR: interquartile range, SCr: serum creatinine.

^a^ Excluded 5 patients in eGFR < 30 group and 34 patients in eGFR ≥ 30 group due to data missing.

^b^ Adjusted by age, sex, COVID-19 disease status, COVID-19 vaccine doses, CCI score, chronic liver disease.

^c^ Excluded 38 patients in eGFR < 30 group and 141 patients in eGFR ≥ 30 group due to data missing.

^d^ Excluded 122 patients on dialysis and 10 patients with data missing in eGFR < 30 group and 31 patients with data missing in eGFR ≥ 30 group.

^e^ Adjusted by age, sex, COVID-19 disease status, COVID-19 vaccine doses, CCI score, the 10 nephrotoxic drugs listed in Table 1.

^f^ Excluded 122 patients on dialysis and 12 patients with data missing in eGFR < 30 group and 31 patients with data missing in eGFR ≥ 30 group.

^g^ Excluded 81 ventilator users in the index date (29 in < 30 group and 52 in ≥ 30 group).

^h^ Adjusted by age, sex, COVID-19 disease severity, COVID-19 vaccine doses, CCI score, chronic lung disease, smoking status, oxygen requirement.

^i^ Adjusted by age, sex, COVID-19 disease status, vaccine, CCI score, hypertension, diabetes mellitus, chronic heart disease, chronic lung disease, chronic liver disease, cancer, cerebrovascular accident, smoking status, and oxygen requirement.

^j^ Excluded patients on the 3-day remdesivir regimen as they did not require supplemental oxygen.

The risk of COVID-19 disease progression was higher in the eGFR < 30 group than the eGFR ≥ 30 group (aOR 1.62, 95% CI 1.16–2.26). After stratifying by remdesivir treatment, only in the 5-day regimen that the eGFR <30 group exhibited a higher risk of COVID-19 disease progression (31.4% vs. 20.6%, aOR 1.73, 95% CI 1.17–2.55]. There was no difference in the length of stay in dedicated wards and the length of oxygen requirement between the two groups.

### Sensitivity analyses

Results were generally consistent across sensitivity analyses. When the CKD-EPI or CG formula was used to calculate eGFR, the risk estimate of all outcomes did not change apparently from those in the main analysis using the MDRD formula (Supplementary Table S1). The incidence of AKI varied depending on the definition used for AKI outcomes. In the eGFR < 30 group, the incidence of AKI was 21.5% according to the KDIGO definition, 8.8% according to the ADQI definition, and 12.7% according to the AKIN definition (Supplementary Table S2). However, the aHRs for AKI remained consistent with the primary analysis, showing an increased risk of AKI in the eGFR < 30 group regardless of the AKI definition used.

## Discussion

In this retrospective cohort study, we discovered that among hospitalized COVID-19 patients receiving remdesivir, those with eGFR < 30 were linked to a significant increase in the risk of AKI, hospital mortality, and COVID-19 disease progression compared to patients with eGFR ≥ 30. There was no difference between the two groups in terms of bradycardia, mortality in COVID-19 dedicated wards, changes in laboratory data, and the time of recovery from COVID-19. To the best of our knowledge, this is the largest study to investigate the safety and effectiveness of remdesivir in patients with an eGFR of < 30 mL/min.

The present study provides insight into the potential safety issues of remdesivir in patients with severe renal impairment, suggesting remdesivir may be used cautiously in this population after weighing risks and benefits. Evidence regarding remdesivir’s safety and effectiveness profiles in other vulnerable groups, such as the elderly, those with multiple comorbidities, children, pregnant/lactating women, and immunocompromised transplant patients, remains limited [37–39]. Future studies should prioritize evaluating remdesivir in these underrepresented populations to ensure proper treatment guidance is available.

The incidence of AKI in patients with eGFR < 30 in our study (21.5%) was notably higher than that reported by Ackley et al. (5%) and Sunny et al. (11%) [14,15], but significantly lower than the 35.2% reported by Wang et al. [17] This discrepancy may arise from variations in the renal function formulas employed and the definitions used for AKI outcomes. Had we applied the same AKI definition, our incidence of AKI would likely have been quite comparable. When utilizing the ADQI criteria, we observed rates of 8.8%, as opposed to Ackley’s 5%, while the use of the AKIN criteria resulted in rates of 12.7%, compared to Sunny’s 11%. In addition, even with the same AKI definition according to KDIGO criteria, there was a significant discrepancy between our study and that of Wang et al. suggesting the characteristics of the patient cohort and the study period may have a certain impact. Recognizing that these operational definitions can significantly impact study outcomes, we conducted sensitivity testing to provide a more profound understanding of the limitations and uncertainties inherent in our findings. This approach allows for a more comprehensive and nuanced interpretation of results, thereby enhancing the reliability and robustness of our conclusions.

We observed a heightened risk of AKI in the eGFR <30 group compared to the ≥30 group, which contradicts the findings of previous literature that reported no significantly increased risk [14,15,20]. This disparity can likely be attributed to the limited sample sizes in those earlier studies, where the number of individuals with AKI in the eGFR <30 group fell below 5, resulting in insufficient statistical power to detect differences.

Furthermore, our study utilized the incidence rate to assess AKI risks, while previous studies relied solely on incidence. The use of both the incidence rate and hazard ratio in our analysis minimizes potential biases when evaluating the risk of drug-related adverse events [40].

Previous studies have identified several risk factors for AKI in COVID-19 patients, including age, male gender, obesity, the use of diuretics and vasopressors, as well as underlying comorbidities, such as CKD, hypertension, and diabetes [41–43]. In our present study, we considered these possible confounders and adjusted them in the statistical models to increase the robustness of the results. We finally observed a higher risk of AKI in the severe renal impairment group. These findings suggest that the use of remdesivir in individuals with severe renal impairment may carry potential renal side effects, necessitating a careful evaluation of the drug’s benefits and risks before its administration. If remdesivir is deemed necessary in patients with severe renal impairment, close monitoring of renal function is imperative. Additionally, early consideration of adjunctive therapies, including dialysis and infusion management, is advisable in the event of declining renal function, while addressing the underlying cause of AKI remains essential.

Our study further revealed that patients with eGFR < 30 had comparable dedicated ward mortality but a higher risk of hospital mortality than patients with eGFR ≥30. A unique aspect of this study was the consideration of death in the dedicated ward as an outcome, which could potentially offer a more accurate measure of remdesivir’s effectiveness. Our hospital mortality rate of 14.1% closely mirrors the WHO Solidarity 2022s rate of 14.5% among all patients receiving remdesivir [44]. Prior investigations into 30-day mortality among remdesivir-treated patients have reported no differences or even higher mortality rates in the eGFR < 30 group compared to the eGFR ≥ 30 group [14,15,20]. However, these studies varied in terms of patient populations, healthcare settings, practices, and study designs, making direct comparisons challenging. Numerous studies have consistently demonstrated that severe renal impairment ranks among the most significant comorbid medical conditions associated with COVID-19-related mortality [4,5].

Moreover, we hypothesize that the increased risk of hospital mortality in patients with an eGFR of <30 is predominantly attributed to their compromised renal function. Our findings highlight the need for vigilant monitoring of clinical parameters, including vital signs and oxygen requirements, in individuals with impaired renal function treated with remdesivir and the need to adjust treatment and medical support accordingly. These results underscore the importance of including this vulnerable population in clinical trials evaluating COVID-19 antiviral therapy.

In our report, the incidence of bradycardia among remdesivir recipients was 19.0%, which falls at the lower end of the 17–74% range reported in other studies [45–49]. The incidence in the cohort with an eGFR <30 was 20.5%, compared to 39% in a small study involving only 31 patients [50]. Our results showed that the risk of bradycardia in the eGFR <30 cohort was comparable to that in the eGFR ≥30 cohort. Current reports have almost exclusively compared remdesivir users with non-users, and limited studies compared the same groups as our condition [51,52]. Furthermore, previous studies have been inconsistent regarding whether renal function may be a risk factor for bradycardia in remdesivir recipients; while Ai et al. suggested that CKD was associated with bradycardia, Hajimoradi et al. found no association between CKD and bradycardia [51,53]. In clinical practice, patients receiving remdesivir should be aware of the potential risk of bradycardia, and appropriate monitoring strategies should be implemented. If bradycardia occurs, prompt intervention should be undertaken to prevent further progression to severe arrhythmias and to ensure the safety of remdesivir recipients.

### Strengths and limitations

The current study has several notable strengths. First, it represents the largest cohort study conducted to date, focusing on the safety and effectiveness of RDV in patients with severe renal impairment. Second, our sensitivity analyses explored various scenarios, bolstering the robustness of our findings. Lastly, we employed rigorous statistical methods to control for potential confounding variables, thereby enhancing the internal validity of our study.

Nonetheless, this research is not without its limitations. First, due to its retrospective observational study design, we cannot entirely rule out the possibility of residual or unmeasured confounding factors. Second, the reliance on data from a single center may restrict the generalizability of our findings to other settings or populations. These limitations necessitate caution when extrapolating and applying the results to broader clinical contexts. More extensive prospective studies are necessary to confirm these issues further.

## Conclusion

Among hospitalized COVID-19 patients treated with RDV, those with severe renal impairment exhibited a heightened risk of acute kidney injury, hospital mortality, and COVID-19 disease progression compared to patients without severe renal impairment.

## Supplementary Material

Supplemental Material

## Data Availability

The datasets created and examined in the present study cannot be accessed by the public due to the regulations of the Institutional Review Board at Taipei Veterans General Hospital. The dataset can be obtained from the corresponding author or IRB of TVGH upon request (email: irbopinion@vghtpe.gov.tw). Data can only be utilized for research purposes.
