# Supplementary material for: Safety and effectiveness of remdesivir in hospitalized patients with COVID-19 and severe renal impairment: experience at a large medical center
Source: Ann Med. 2024 Jun 3;56(1):2361843. doi: 10.1080/07853890.2024.2361843 (PMC11149583; doi:10.1080/07853890.2024.2361843)
Supplement: Supplemental Material [file IANN_A_2361843_SM6390.docx]

**Supplementary Table 1. Sensitivity analysis by grouping of different renal function formulas**

|  | aHR/aOR/β (95% CI) | | |
| --- | --- | --- | --- |
| Outcome | MDRD formula | CKD-EPI formula | Cockcroft-Gault formula |
| **Safety Outcomes** |  |  |  |
| Acute kidney injury^a^ | **2.92 (1.93–4.44)** | **2.61 (1.73–3.93)** | **1.65 (1.08–2.52)** |
| Bradycardia^b^ | 1.15 (0.85–1.56) | 1.15 (0.86–1.55) | 0.96 (0.73–1.26) |
| Laboratory data change |  |  |  |
| ALT^d^ | 7.20 (-36.13–50.51) | 10.90 (-31.46–53.26) | 4.31 (-35.34–43.96) |
| Total bilirubin^d^ | -0.08 (-0.34–0.18) | -0.06 (-0.32–0.20) | -0.09 (-0.33–0.16) |
| eGFR^a^ | 1.99 (-1.58–2.26) | 1.84 (-0.39–4.07) | 1.38 (-0.72–3.49) |
| SCr^a^ | 0.08 (-0.04–0.19) | 0.04 (-0.07–0.15) | -0.03 (-0.12–0.07) |
| **Effectiveness outcomes** |  |  |  |
| Mortality in dedicated ward^c^ | 1.43 (0.90–2.23) | 1.30 (0.82–2.06) | 1.19 (0.76–1.85) |
| Progression of COVID-19 disease^e^ | **1.62 (1.16–2.26)** | **1.66 (1.20–2.29)** | **1.46 (1.07–1.99)** |
| 3-day remdesivir regimen | 1.34 (0.68–2.61) | 1.03 (0.52–2.02) | 1.13 (0.60–2.14) |
| 5-day remdesivir regimen | **1.73 (1.17–2.55)** | **1.92 (1.31–2.82)** | **1.59 (1.11–2.27)** |
| Length of stay indedicated ward^c^ | -0.08 (-0.97–0.81) | -0.08 (-0.95–0.79) | 0.55 (-0.24–1.33) |
| Length of oxygen requirement^e^ | 0.34 (-1.92–2.60) | 0.33 (-1.88–2.54) | -1.15 (-3.23–0.94) |

aHR: adjusted hazard ratio, aOR: adjusted odds ratio, CI: confidence interval, CKD-EPI: Chronic Kidney Disease Epidemiology Collaboration, MDRD: modification of diet in renal disease

^a^ Adjusted by age, sex, COVID-19 disease severity, COVID-19 vaccine doses, CCI score, the 10 nephrotoxic drugs listed in Table 1

^b^ Adjusted by age, sex, COVID-19 disease severity, COVID-19 vaccine doses, CCI score, hypertension, diabetes mellitus, chronic heart disease, cerebrovascular accident

^c^ Adjusted by age, sex, COVID-19 disease severity, COVID-19 vaccine doses, CCI score, hypertension, diabetes mellitus, chronic heart disease, chronic lung disease, chronic liver disease, cancer, cerebrovascular accident, smoke status, oxygen requirement

^d^ Adjusted by age, sex, COVID-19 disease status, COVID-19 vaccine doses, CCI score, chronic liver disease

^e^ Adjusted by age, sex, COVID-19 disease status, COVID-19 vaccine doses, CCI score, chronic lung disease, smoking status, oxygen requirement

**Supplementary Table 2. Sensitivity analysis of acute kidney injury definition**

|  | eGFR < 30  n = 185 | eGFR ≥ 30  n = 1,036 | Adjusted HR^a^ (95%CI) |
| --- | --- | --- | --- |
| KDIGO, n (%) | 39 (21.5) | 76 (7.6) | **2.92 (1.93–4.44)** |
| ADQI, n (%) | 16 (8.8) | 56 (5.6) | 1.64 (0.91–2.95) |
| AKIN, n (%) | 23 (12.7) | 22 (2.2) | **4.99 (2.71–9.19)** |

ADQI: Acute Dialysis Quality Initiative, AKIN: Acute Kidney Injury Network, eGFR: estimated glomerular filtration rate in mL/min/1.73 m^2^, KDIGO: Kidney Disease: Improving Global Outcomes,

^a^ Adjusted by age, sex, COVID-19 disease severity, COVID-19 vaccine doses, CCI score, the 10 nephrotoxic drugs listed in Table 1
